# Dropout and transfer paths: What are the risky profiles when analyzing university persistence with machine learning techniques?

**DOI:** 10.1371/journal.pone.0218796

**Published:** 2019-06-21

**Authors:** Luis J. Rodríguez-Muñiz, Ana B. Bernardo, María Esteban, Irene Díaz

**Affiliations:** 1 Department of Statistics, O.R., and Mathematics Education, University of Oviedo, Asturias, Spain; 2 Department of Psychology, University of Oviedo, Asturias, Spain; 3 Department of Computer Science, University of Oviedo, Asturias, Spain; IUMPA - Universitat Politecnica de Valencia, SPAIN

## Abstract

University dropout is a growing problem with considerable academic, social and economic consequences. Conclusions and limitations of previous studies highlight the difficulty of analyzing the phenomenon from a broad perspective and with bigger data sets. This paper proposes a new, machine-learning based method, able to examine the problem using a holistic approach. Advantages of this method include the lack of strong distribution hypothesis, the capacity for handling bigger data sets and the interpretability of the results. Results are consistent with previous research, showing the influence of personal and contextual variables and the importance of academic performance in the first year, but other factors are also highlighted with this model, such as the importance of dedication (part or full time), and the vulnerability of the students with respect to their age. Additionally, a comprehensive graphic output is included to make it easier to interpret the discovered rules.

## Introduction

Governments have promoted the democratization of access to higher education systems, and this broader access has increased interest in dropout at the university level. Research on this topic grew in parallel with the development of statistical techniques and computing capacity. The literature presents many studies analyzing the causes of dropout and establishing models for detecting, predicting and preventing it. Nevertheless, researchers must remember that different definitions of dropout have been applied. Throughout this paper, we use the definition of dropout used in the Spanish context, described by the National Agency for Quality, acknowledging students as having withdrawn when, after being registered in a particular program, they do not enroll again for the following two years.

In Spain, the European Higher Education Area and University 2020 framework have led to a major concern in universities regarding student affairs services. Tutorial action plans have been implemented in most faculties since 2010, and attention has also focused on soft skills, cross-curricular issues, and complementary activities, in addition to greater monitoring of students’ academic paths [1]. Within the European Higher Education Area dropout is still not very deeply studied but it is a topic of some concern; the Europe 2020 strategy states dropout reduction rates for every educational stage that members should have achieved by this date.

In [2] five different explicative approaches to dropout phenomenon are distinguished: psychological, sociological, economic, organizational, and the interactionalist approach, proposed by Tinto [3, 4]. Other authors also consider a sixth approach, called integrationist or holistic, with different stages for different sets of variables that can influence dropout phenomena [5]. This approach considers the influence of all of the above approaches, connecting prior and current academic experiences. Because of this, student affairs and services are considered fundamental to prevent dropout [6]. This perspective highlights the fundamental role of relationships between members of the educational community, students, teachers, administration officers, families, stakeholders and others that can potentially work together, building a healthy educational environment. Given the high cost of dropout not only to students and their families, but also to universities and governments, it is particularly important to properly transfer research findings to stakeholders in order to reduce the phenomenon, as all educational research should aim to [7]. Consequently, studies like one presented here are very important tools to reduce dropout rates and to mitigate the dangerous consequences of this educational problem [8,9].

Apart from the explicative approach, researchers have used different types of data analysis methodologies. Common techniques include correlational analysis [10–13], univariate or multivariate variance analysis [14], logistic regression and structural equations [15–18], and multilevel analysis [19]. The main difficulties applying statistical techniques arise from the required hypothesis for the data (such as normality) and from the difficult interpretation of discovered relationships, especially for a non-specialist audience. Correlations, regression coefficients and factorial analysis are a specific jargon which makes the interpretation of the results by stakeholders much harder. Interpretability is particularly important when transferring research results to the education community. To help in improving interpretability, machine learning (ML) methods [20] and data mining techniques [21] are considered good tools, and they also ease the required conditions for data.

Although Educational Data Analysis (EDA) is gaining importance as a research topic, few studies apart from [22] can be found applying EDA within the context of dropping out looking at the entire set of degree courses in a university. More studies can be found applying EDA to specific fields such as a limited set of university degree courses [23], courses in open or online universities [24], e-learning courses [25–27], and MOOCs [28]. Within EDA there are various techniques based on ML, which is a branch of artificial intelligence providing methods with the ability to learn from data and/or to make predictions or classifications [29].

Both statistics and ML share the common objective of learning about underlying phenomena by using previously generated data. However, they are quite different approaches. Statistics is usually based on certain assumptions. For instance, a linear regression assumes a linear relationship between an independent and a dependent variable, homoscedasticity and independence of observations. ML algorithms, on the other hand, do need some conditions to be met but in general, are spared from most of the statistical assumptions. The lack of assumptions about the data structure allows ML algorithms to construct complex non-linear models, which are much softer than statistical models. Another advantage of ML is based on the types and quantity of data it deals with. There are some ML tools that are exceptionally fast with big data (both large numbers of attributes and large numbers of observations). Another advantage of ML tools is their interpretability. They are easier to understand than other more mathematical-based statistical tools.

There are many different paradigms in ML: lazy methods such as KNN [30], methods based on tree construction such as C4.5 [31], classification and regression trees [32], or Neural and Bayesian networks [29]. In predicting university dropout, the majority of research using ML techniques has tried to predict students’ grades or their persistence on courses (mostly e-courses), but massive data from all the degree courses in one university are not found in the literature, except in [22].

The aim of the present study is to undertake the analysis of dropout of a full cohort of students in the University of Oviedo (Spain) by using ML-based methods to extract rules helping stakeholders to identify dropout and attrition paths, so that they can predict the phenomenon before it happens, and to prevent dropout by taking appropriate measures to increase persistence in university. Our goal is to find a model that can be exported to other universities, identifying common characteristics of dropout behavior, and also discovering new relationships between variables.

We propose the following research hypotheses:
RH1: By using ML techniques it will be possible to extract rules that can predict university dropout for an entire cohort of students on different degree courses.
RH2: Rules will define dropout paths combining students’ personal, academic and non-academic characteristics, as well as environmental variables
RH3: Some of the rules will reveal associations between variables which had either remained hidden in previous research or were much more difficult to interpret when using classical statistical techniques.
RH4: The computational efficiency of the new method will improve on results obtained using previous approaches.

## Method and materials

This section describes the methodological framework used in this work.

### Population and sample

The population is the cohort of freshmen in academic year 2010/11 in a medium-sized university (University of Oviedo) in the Spanish region of ASTURIAS. This university offers 54 bachelor degrees, and has approximately 28500 people associated with it, made up of 25000 students (around 20000 undergraduates), 2500 teaching staff, and 1000 administration and other service officers. The cohort being studied is particularly interesting since it was the first to take part in the Bologna process. That year, 5215 students entered the institution, two years later (the beginning of academic year 2012/2013), 4149 students were still on their initial degree course, 363 had transferred to another degree in the same university, and 658 had left the university. It is important to note that this university is the only university (either public or private) in the region of ASTURIAS (Spain), so for those students living near the university, dropping out also means moving away from home.

As the first stage of data collection, the following socio-academic information was extracted from the institutional data store: identification data, sex, birthplace, nationality, disability, family size, parents’ educational qualifications and current occupations, average score in previous education, score in university admission exam, age when admitted, date of first enrolment, priorities stated on the course admission application, knowledge area corresponding to the student´s course, number of enrolled credits, passed credits and average score, whether they have a grant or scholarship, current academic situation and, where there is a program or university transfer, the destination.

To provide more information about students’ contexts and situations other data were necessary. Hence, a random stratified sampling was selected to complete an *ad hoc* questionnaire, after confirming the impossibility of polling the whole population due to cost constraints and because it was difficult to get in touch with many students that had dropped out. Since questionnaire response rates are generally low when polling dropped-out students, a nonprobabilistic intentional sampling was performed until acceptable rates were reached in each of the strata and blocks explained below.

Spanish university degree courses are classified into five knowledge areas, which guided a random stratified sampling to obtain a sample of N = 1055 students, divided into two sub-samples, those who have stayed in the same program (N = 626) and those who have dropped out from it (N = 429). Additionally, quotas were established in the dropout sub-sample distinguishing those who transferred to another degree in the same institution (276) and those who left the institution (153). Table 1 shows the distribution of the sample.

**Table 1:** Sample distribution by knowledge area.

Knowledge area	Stay		Change		Dropout		Total	
Arts & Humanities	62	5.88%	14	1.33%	43	4.08%	119	11.28%
Engineering & Architecture	171	16.21%	51	4.83%	84	7.96%	306	29.00%
Health Sciences	83	7.87%	7	0.66%	17	1.61%	107	10.14%
Law & Social Sciences	258	24.45%	69	6.54%	111	10.52%	438	41.52%
Sciences	52	4.93%	12	1.14%	21	1.99%	85	8.06%
Total	626	59.34%	153	14.50%	276	26.16%	1055	100%

Percentages in each cell are calculated from the total number of cases

### Reseach design and instruments

The study used an *ex-post facto* design, using a holistic approach to analyze the dropout phenomenon, acquiring university data, complemented by an *ad hoc* questionnaire (available at https://goo.gl/5t09wB). This questionnaire was completed either over the telephone or by email by all the individuals in the sample (N = 1055). Items were related to:

- Marital status, income level, type of housing during the course.
- Motivations for choosing the program and this university.
- Participation in welcome activities for freshmen and opinion of them.
- Time spent on study, work, and housework.
- Assessment of program requirements, and satisfaction with scores.
- Evaluation of personal relationships (teachers and peers).
- Dropout intention, and reasons, if it happened.
- If dropped-out, current situation and degree of satisfaction with the results of the decision.
- Degree of satisfaction with the University of Oviedo.

The questionnaire was composed of closed multiple-choice questions with between 3 and 8 answers, closed questions with Likert-type scales, and a few open questions. Since we assumed a holistic study framework, in addition to academic variables, we also included others related to student educational and socioeconomic background and current situation, motivation, engagement, personal relationships within the institution, and indicators of satisfaction with the institution.

### Procedure

The data collection was carried out through two complementary processes; request of basic information from the university administration and application of the ad hoc questionnaire (by email or phone). We considered social and demographic variables (age, civil status, place of residence, gender, number of family members, nationality, presence of a disability, non academic work and housework hours per week, parents’ educational qualifications, etc.), academic variables (access path to university, overall grade in secondary school, ‘Why did you choose the University of Oviedo?’, ‘Why did you choose your degree course?’, ‘Did you receive guidance before entering University?’, participation in integration activities, class attendance, whether students perceive the university methodology to be appropriate to the course, number of study hours per week, grades-effort ratio, difficulty of the degree, relationship with teachers and peers, enrolling in non-academic activities, number of enrolled, attended and passed credits in the previous year, etc.), and economic variables (family income, parents’ occupation, having a grant in the current year, etc.).

### Modelling dropout as an ML problem

Once the information was gathered, next step was to model withdrawal as an ML problem. Thus, starting with a sample population consisting of *n* observations (*n* = 1055, in this sample) belonging to *C* classes (*C* = 3, *Dropout*, *Change* and *Continue* classes), an ML technique constructs a model to predict these *C* categories and, at the same time, it tries to identify factors characterizing the different withdrawal profiles.

There is no ML method that can be selected as the best one beforehand. Thus, the baseline selected method was identified after comparing different performances from several techniques. In particular, we tested different kinds of machine learners for this problem: C4.5 [31], Random Forest [33], CART [34], Bayes Nets [35], and Support Vector Machines (SVM) [36,37].

Some of the most challenging yet fruitful ML approaches are those based on tree models, due to their performance and good interpretability. Tree-based models build a tree using forward selection by a top-down approach from root to leaves until some stopping condition is reached. At each step, they find the best split according to some impurity measure. The node associated with maximal impurity reduction is then selected. C4.5 and CART are examples of this paradigm.

Bayesian network classifiers are a special type of Bayesian network designed for classification problems. They offer an explicit, graphical, and interpretable representation of uncertain knowledge. Their semantics are based on the concept of conditional independence. As they output a probabilistic model, decision theory is naturally applicable to dealing with cost-sensitive problems, thereby providing a confidence measure on the chosen predicted label [38].

Finally, SVM is an ML approach that constructs a hyperplane or set of hyperplanes in a high-dimensional space. Good separation is achieved by the hyperplane having the largest distance from the nearest training-data point of any class. Often, the sets to discriminate are not linearly separable in the initial space. For this reason, the original finite-dimensional space is often mapped into a much higher-dimensional space, making the separation easier in that space.

## Results

First, we include a statistical description of the attributes, in order to provide a general overview of the values in the problem. Table 2 gives the main values of the quantitative attributes, while in Table 3 the modes of qualitative attributes are shown (we have omitted all the frequency tables for clarity).

**Table 2:** Statistical description of quantitative attributes.

Attribute	Mean	SD	Median	Min	Max
Number_FamilyMembers	3.52	0.99	4	0	8
StudyHours_PerWeek	11.76	8.29	10	0	60
WorkHours_PerWeek	4.04	10.07	0	0	81
HouseWorkHours_PerWeek	4.98	6.21	4	0	70
Age	20.33	5.27	18	17	63
BACH_FP	5.08	3.35	6.12	0	10
GEN	4.12	3.33	5.25	0	9.76
Grade	5.62	3.64	6.29	0	13.66
Area_Code	3.70	1.27	4	1	5
Enrollment_Credits_2010	57.92	7.27	60	6	72
Attended_cred_2010	45.71	19.04	54	0	72
Passed_Credits_2010	32.21	23.10	36	0	72
Enrollment_Credits_2011	35.59	30.53	54	0	90
Attended_credits_T_2011	31.96	28.79	42	0	84
Passed_Credits_2011	25.89	26.00	21	0	78

**Table 3:** Mode of qualitative attributes.

Qualitative Attribute	Mode
Place_of_Residence	Family house
Studies_Choice_reason	Vocation
Prior_guidance	No
Integration_activities_utility	Enough
Participation_Integration_Activities	No
Class_Attendance	Very Much
Appropriate_methodology	Sufficient
RatioGradesEffort	Satisfactory
Difficulty	High
Teacher_Relation	Close enough
JoinNonAcademic_activities	Nothing
Would_recommend_uniovi	Sufficient
Gender	Woman
Nationality_code	Spanish
Disabilability_Code	No
Fam_Code	No
Father_Stud_Code	2
Mother_Study_Code	2
Father_job	EO
Mother_job	Unknown
Acces_Way	University entrance exams
Center_Code	University
Priority	0
First_Year_Grant_Code	No
Grant_2011	No

**Fig 1:**
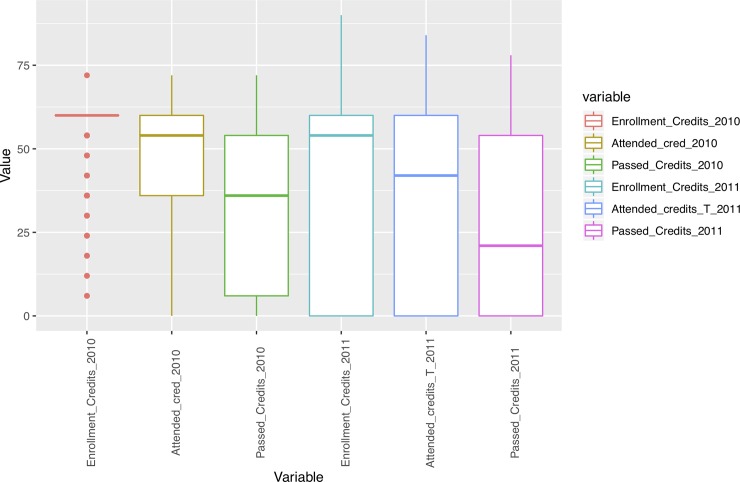
Box-plots for variables concerning number of credits.

**Fig 2:**
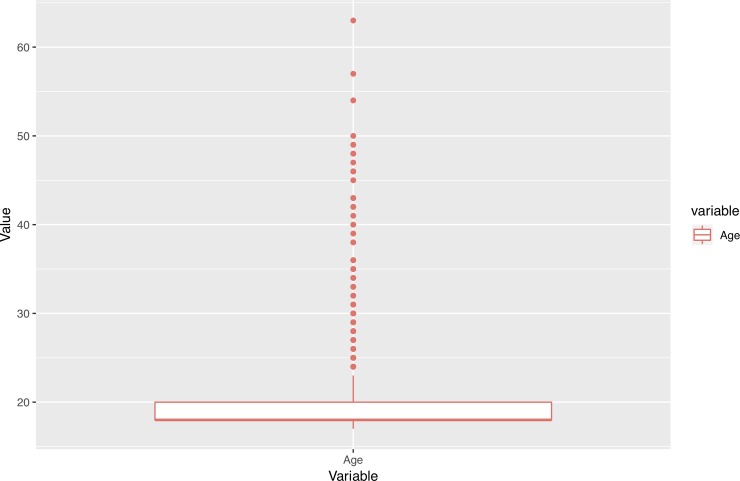
Box-plots for age.

**Fig 3:**
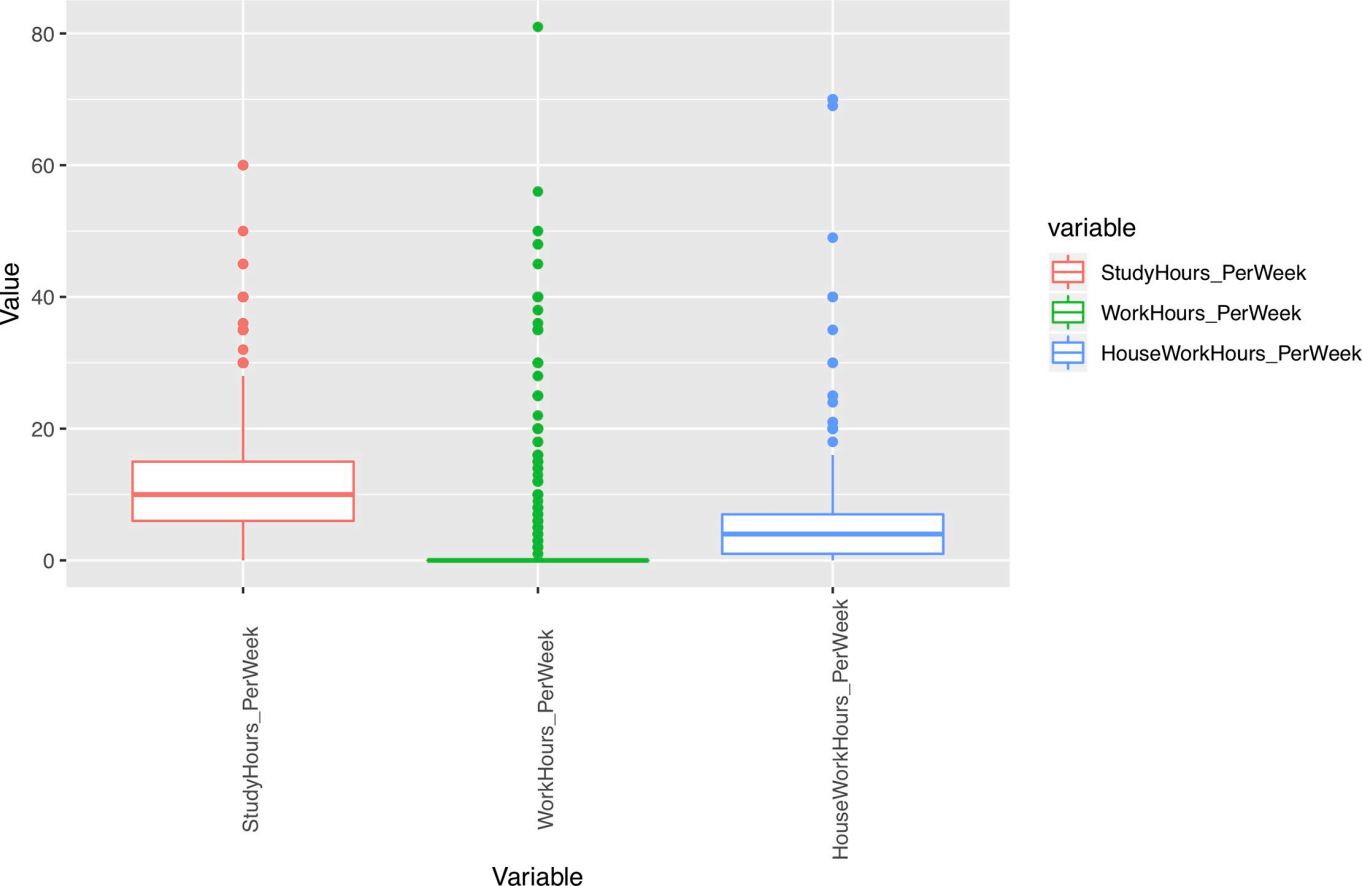
Box-plots for variables concerning time.

**Fig 4:**
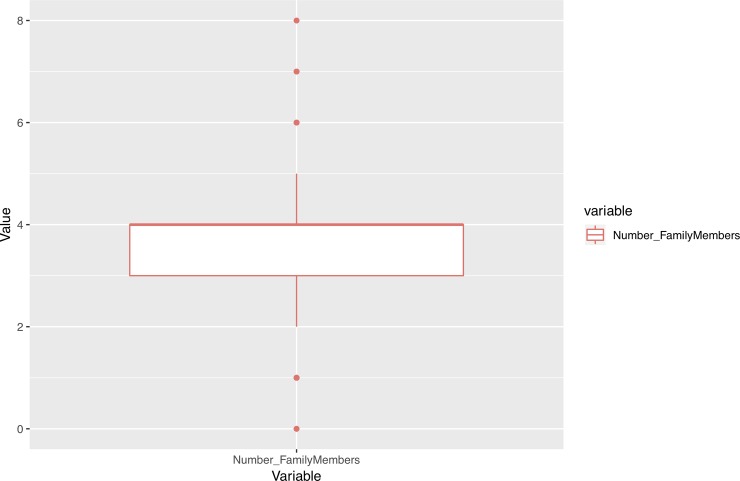
Box-plots for number of family members.

**Fig 5:**
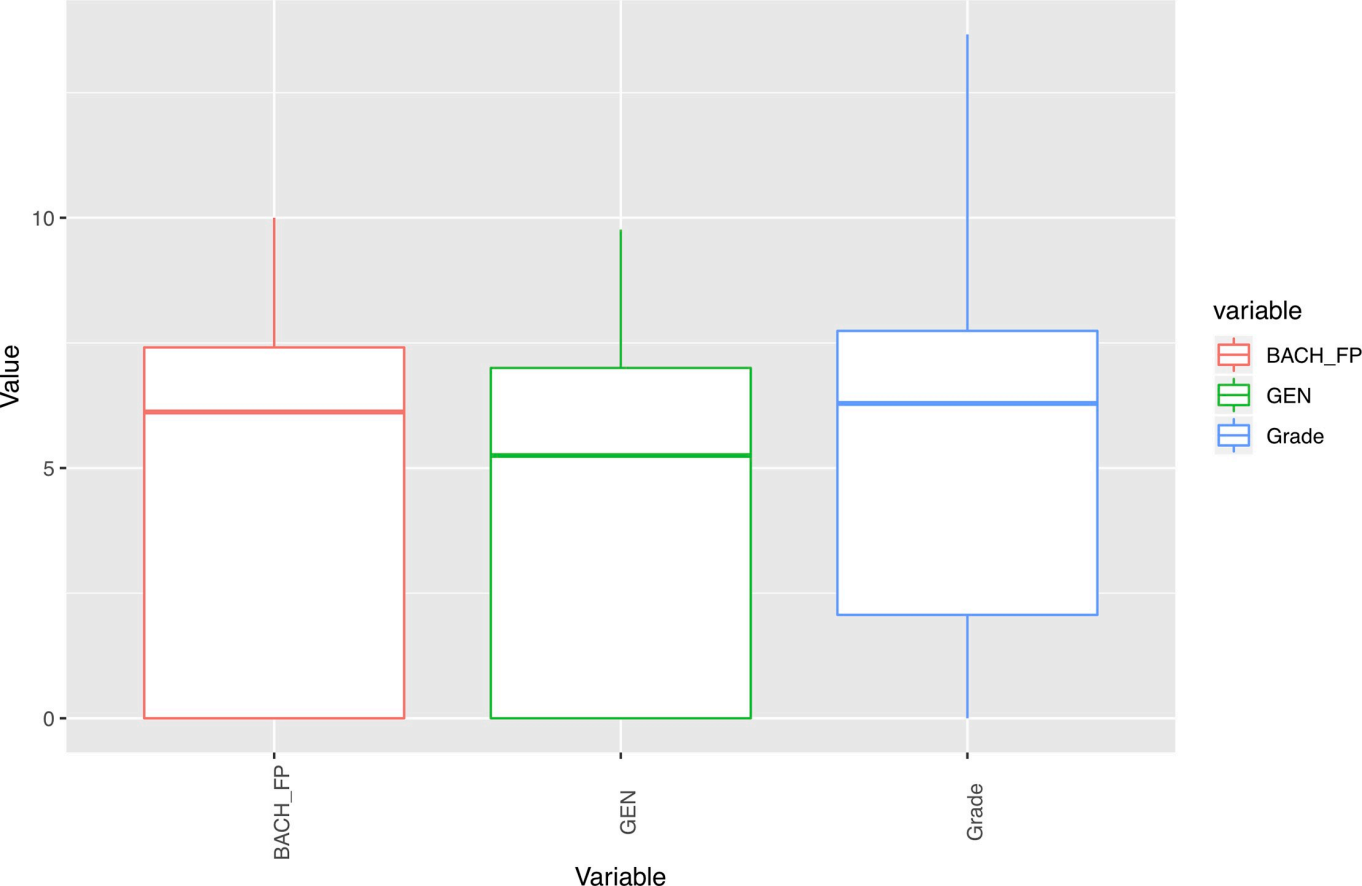
Box-plots for variables concerning academic records.

In addition, we performed an outlier analysis of variables in order to determine possible influences of extreme values on the results. As Figs 1–5 show, the variables do not generally present outliers. In the case of number of enrolled credits in 2010 (Fig 1) we see that there is a remarkable concentration of cases in 60, because academic policies at that time forced incoming students to enroll for 60 credits, with few exceptions (depending on personal situation, background, etc.) which explain the other observed values. Nevertheless, they cannot be considered outliers, but it is simply a very concentrated variable with a strongly skewed distribution. A similar situation occurs with age (Fig 2), but as will be explained later, the rule concerning age has a turning point at 23 years old, and therefore even when there are some older students, it does not affect the output. Other skewed variables such as number of working and houseworking hours per week (Fig 3) do not influence the rules obtained, therefore their outlier analysis is irrelevant.

**Fig 6:**
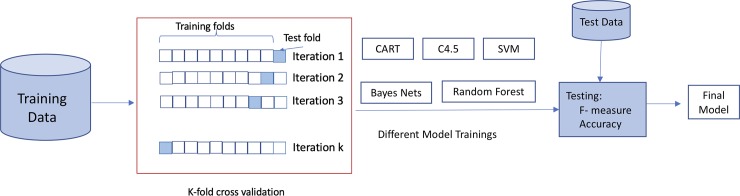
Scheme of the procedure.

Different combinations of metrics, splits, stopping conditions and pruning methods lead to different approaches when using ML techniques to construct classification tree models. Thus, experiments were run using the RWeka package, version 0.4–36. In this study, we tested all the machine learners mentioned in Subsection 2.4. All were trained using their default configurations. Additionally, all the classifiers were trained by using cross-validation, with 10 bins, and by repeating the experiments 10 times. In the testing step, the performance of these classifiers was measured using the well-known measures Precision, Recall, *F*-measure and Accuracy [39]. Precision is the fraction of relevant examples in the retrieved instances. Recall is the fraction of relevant instances that have been retrieved over the total amount of relevant instances. The *F*-measure, defined as the harmonic mean between Precision and Recall, is quite a common measure for weighting the well-known existing trade-off between Precision and Recall. The *F*-measure, together with Accuracy (defined as the percentage of success), are the commonly-used tools for evaluating the classifiers. The general scheme of the process followed in this study is given in Fig 6.

Table 4 shows the performances of the different methods with default configurations. They were quite similar, no significant differences were found between the methods. C4.5 is slightly better than other methods, except SVM, in terms of *F*-measure and slightly worse than all the methods, except CART, in terms of Accuracy. However, it is well known that Accuracy is not a good performance measure for unbalanced datasets, like ours (626 students continuing, 153 dropping out and 276 transferring). As we were looking for a method that was easy to understand, C4.5 was finally selected as the best option for predicting drop-out.

**Table 4:** Performance of each method for the total sample.

Sample	Method	*F*-measure	Accuracy
Total sample	CART	82.7%	83.5%
	C4.5	85.0%	85.2%
	Bayes Nets	83.7%	86.2%
	Random Forest	84.7%	86.6%
	SVM	85.6%	85.5%

Note that each individual in the sample was characterized by the dropout behavior with respect to the previously-defined 46 variables. Therefore feature selection was also performed to check whether it produces a clearer model or not. Furthermore, it is also well known that the pruning process can be supervised by using the onfidence factor (*c*) and the minimum number of examples per leaf (*minNumObj*) in C4.5. The default *c* is .25 and the default *minNumObj* = 2. The classifier was tested with confidence factors ranging from .05 up to 1, to obtain the optimal pruned C4.5 tree. The minimum number of examples per leaf was also tested. For feature selection, the gain ratio was used as an attribute evaluator to filter at 50% and 25% levels. Table 5 shows the performance of some combinations measured by *F-*score. Values for *c* below .1 and for *minNumObj* over 10 make the performance worse. At the same time, too-aggressive feature selection also leads to a worse learning process.

**Table 5:** Performance of C4.5 for the whole sample.

C4.5	*F*	*TREE SIZE*
*c* = .25, minNumObj = 2	85.0%	75
*c* = .25, minNumObj = 10	84.9%	39
*c* = .1, minNumObj = 2	84%	46
*c* = .1, minNumObj = 10	83.6%	5
FS– 50%	83.2%	81
FS– 25%	83.5%	5

Data in Table 5 demonstrate that different configurations of C4.5 have almost the same performance in terms of *F*-measure, although feature selection slightly reduces both performance measures. On the other hand, the tree size varies from 75 to 5 nodes with different pruning strategies. Model complexity is greatly reduced when pruning is aggressive. Therefore, considering both classifier performance and complexity, the selected configuration of C4.5 was setting the confidence factor to *c* = .25, *minNumObj* = 10 and no additional feature selection.

As this problem is quite imbalanced, it is important to also examine associated performance for each class. These results are detailed in Table 6.

**Table 6:** Performance for each class in the sample.

C4.5 with *c* = .25, *minNumObj* = 10	*F*
Change	44.4%
Continue	99.6%
Dropout	72.0%

An initial consideration must be highlighted when looking at Table 6. While classes *Continue* and *Dropout* have a high performance level, the *Change* (transfer) class is much more difficult to correctly predict. When looking at the individuals in this class, we see that most students transfer to another program within the same knowledge field. This can be explained by the structure of the degrees in the University of Oviedo. In the case of engineering degrees (29% of the sample) and most social science degrees (41.52% of the sample), the first year is common to different degree courses although students are enrolled on a specific one; this makes it easier for students to change from one degree to another after their first year. The change is not necessarily forced, and that makes it harder to differentiate those students from students who are forced into changing courses. Therefore, whereas dropout can be forced if students do not achieve a minimum of passed credits (institutional persistence policy), in the case of the *Change* class we find many different profiles: students changing because they do not comply with this policy, students changing after having complied with this policy but who still have a low number of passed credits, and students changing after passing all the credits in their first year simply because they changed their mind, and enroll on a different degree course. This situation and its prevalence in the sample explains the low performance when predicting the *Change* class.

**Fig 7:**
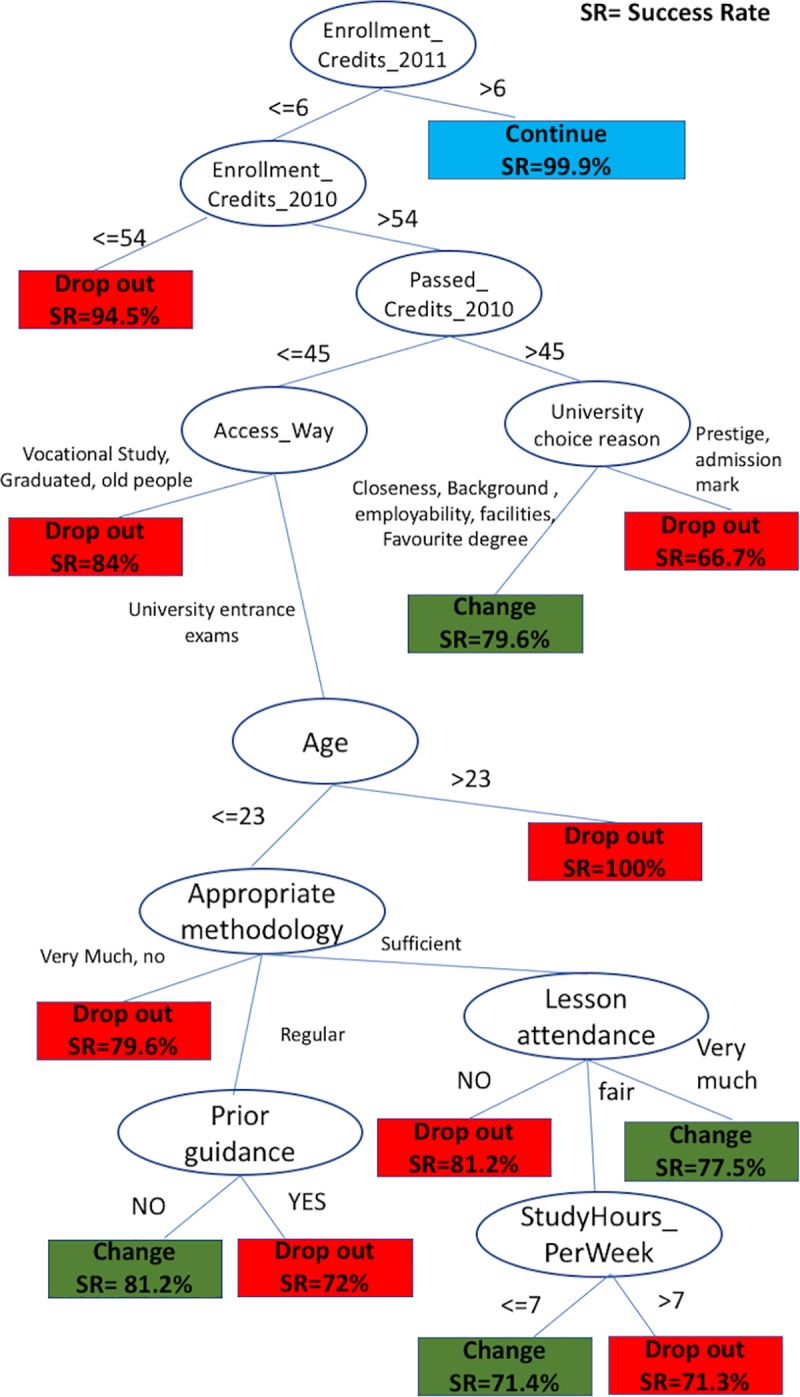
Decision tree to predict drop out or transfer paths.

After this initial consideration, the output tree with relevant variables for dropout, transfer or persistence are displayed in Fig 7 (numbers at the bottom of each leaf show the probability of the leaf, if the leaf displays SR = 72% this means that the probability of finding an individual in the sample satisfying all the characteristics in the path from the root to this leaf is .72).

As Fig 7 shows, predicting continuity is a much easier task than predicting change of degree course or dropout, because the first difference is based on the number of enrolled credits in the second academic year, making continuing in the university very likely if they were enrolled in more than 6 credits. On the other hand, when students are enrolled in 6 credits or less the situation changes dramatically, and continuity becomes much more difficult to achieve.

At the second level of the tree there is a gap between freshmen having taken 54 credits or less in the first year (mainly part-time students, due to academic policies) and those having taken more than 54 credits (generally full-time students, since credits are taken in multiples of 6, so the next step is 60 credits, as displayed in Fig 1). When students take 6 credits or less in their second year, having taken 54 or less in the first year, they will very probably dropout, the chances of dropping out being 95%. This probability is reached by considering only the two aforementioned variables, which clearly shows it is a very risky profile for dropping out.

When students have taken more than 54 credits in their first year, more situations can be distinguished. For these students, the first difference is based on the number of passed credits in the first year. Those passing more than 45 credits can be classified according to why they chose the University of Oviedo. When the reason for the decision is the admission grade or the prestige of the University, the students are much more likely to dropout than when the reason is based on proximity, employability rates, facilities or the type of degree. In the latter case, these students are more likely to transfer to a different degree rather than drop out.

For those students passing 45 credits or less in the first year, the route into accessing higher education becomes relevant. Students could enter university from high school, from vocational training, by holding a previous university degree or for mature students, by passing some specific exams. These last three groups are more likely to drop out than the first. Students in this situation coming from vocational training, graduates from other degrees, or from exams for mature students, will probably dropout (84% chance). Being older is also a risk factor for freshmen coming from high school, since the next branch of the tree shows how students over 23 years old are very likely to drop out (actually 100% in the sample did). These two factors clearly underline age as a very important dropout factor, dramatically increasing the probability of dropout.

Following the branches of the tree down, we arrive at younger freshmen (under 23) coming from high school. In this case, other academic factors appear now, such as the appropriate methodology, class attendance, guidance received prior to enrollment and the number of study hours per week, as it shown in Fig 7. When these students consider the teaching methodology in the degree to be less than adequate they tend to drop out (79.6%). Additionally, when these students consider the methodology to be only acceptable, having received prior guidance comes into play; when they have received prior guidance, they tend to dropout (72%) but when they have not received guidance they tend to transfer to another degree (81.2%). Lastly, when these students consider the methodology to be appropriate, class attendance comes into play; when they do not attend class they tend to dropout (81.2%), when their attendance is high, they tend to transfer to another degree (77.5%) but when the class attendance is fair, study hours play a determining role: so, when students study 7 hours or less per week, they transfer (71.4%) but when they study more than 7 hours, they tend to dropout (71.3%).

Rather than describing the full tree, it is more interesting to identify some risk profiles, following the path from the root to the corresponding leaf on the tree. In our opinion, this is one of the best outputs that the ML approach produces, due to its ease of interpretation.

In addition to the profiles described above, the following are also profiles at high risk of dropping out:

- Students who had taken more than 54 credits in their first year, passing 45 or less, having taken no more than 6 credits in their second year, entering from high school, 23 years old or younger, who think the methodology followed at university is sufficient and who do not attend lectures. They have a dropout rate of 81.2%
- Students who had taken more than 54 credits in their first year, passing 45 or less, having taken no more than 6 credits in their second year, entering from high school, 23 years old or younger, who think the methodology followed at university is either absolutely appropriate or not appropriate. Their dropout rate is 79.5%.
- Students who had taken more than 54 credits in their first year, passing 45 or less, having taken no more than 6 credits in their second year, entering from high school, 23 years old or younger, who think the methodology followed at university is just adequate and who received guidance prior to their enrolment at university. Their dropout rate is 72%.
- Students who had taken more than 54 credits in their first year, passing less than 45, having taken no more than 6 credits in their second year, entering from high school, 23 years old or younger, who think the methodology followed at university is sufficient, have fair lecture attendance and study more than 7 hours a week. Their dropout rate is 71.3%.

We can also highlight the following transfer profiles:

- Students who had taken more than 54 credits in their first year, passing 45 or less, having taken no more than 6 credits in their second year, entering from high school, 23 years old or younger, who think the methodology followed at university is only fairly appropriate and who had received guidance prior to their enrolment at university. Their change rate is 81.2%.
- Students who had taken more than 54 credits in their first year, passing 45 or less, having taken no more than 6 credits in their second year, entering from high school, 23 years old or younger, who think the methodology followed at university is sufficient and who have high lecture attendance levels. They have a change rate of 77.5%.
- Students who had taken more than 54 credits in their first year, passing 45 or less, having taken no more than 6 credits in their second year, entering from high school, 23 years old or younger, who think the methodology followed at university is sufficient, and who have fair lecture attendance and study 7 hours or less a week. Their change rate is 71.4%.

## Discussion

The ML rules extracted which predict dropout share a common core of factors with those produced by statistical methods in previous research, but this current study also introduces new factors and highlights their importance in comparison with previous models.

We saw the importance of performance, but while other studies were much more focused on prior grades [11,17], in our model the most important determining factor is the performance in the first year. This result underlines the importance of the first year in university and challenges higher education institutions to pay special attention to performance in students’ first semesters in order to adopt preventive measures. This attention during the first year has also been remarked on by [40–42].

Lecture attendance is also an important factor, especially for students entering from high school. Not attending or attending few lectures increases the risk of dropout, whereas attending all or most lectures decreases that risk and encourages program transfer instead of university withdrawal. These findings agree with those from other authors [2,40,43,44]. When we consider this result together with freshmen academic performance in our model it highlights the need to reinforce real-time information systems to guide institutional intervention, to detect and prevent possible dropout before it happens [45,46].

The method introduced in this paper also detects other factors which increase the risk of dropout that have not been sufficiently highlighted in previous studies. The first is being a part-time student: The results clearly show how part-time students are more likely to drop out and need to be supported not only at enrolment but also during their courses, making it easier for them to juggle other commitments (such as family or employment). In addition, being a part-time student combined with age (entering university at 23 or older) makes persistence much more difficult.

Differences based on admission profile are another important issue revealed by our analysis. In the Spanish university system, students can enter university from high school (around 75% of freshmen), from vocational education (around 10%), by special exams for mature students (around 4%), or by having a previous degree (4%). The most important contingent comes from high school, meaning that is where universities focus most of their guidance effort. However, the data analysis shows that students entering university by other means are at a much higher risk of dropout than students from high school. This should lead universities to design and develop specific guidance strategies and tutorship for students from vocational education and for mature students.

A third new notable factor extracted from the data is the reason for selecting the particular institution. There is, to some extent, a situation that could be compared to a captive market, which comes from the possibility of doing a similar degree at another university, and the ability to move from the family home. When students choose the University of Oviedo due to their admission scores or employability rates, they are more likely to dropout than when they choose the University of Oviedo because of its proximity. When they choose this university because of proximity, more factors become involved in the dropout decision, and students are more likely to transfer than dropout. In our opinion, the underlying reason is that they do not have the same opportunities to move away when dropping out and so they consider transferring to a different course within the same university. This proves the importance of economic issues when choosing a university course. Although the effect of financial aid has been investigated [47], more data needs to be collected to analyse the effect of students’ initial financial situations, and the relationship with the different kinds of financial support [45,48,49] requires a detailed evaluation that is beyond the possibilities of this study.

Since prediction was one of the main goals of this study, it is very important to quantify how good the prediction is. The accuracy, recall, and *F*-measure for the method we used were discussed in Section 4. Now we want also to underline its importance, by comparing it with some of the previous classification studies in the references. In Table 7 we collect these results, looking at the *F*-measure in the case of supervised classification or prediction methods (such as the decision trees proposed in this study), whereas in other types of methods such as regression the percentage of variability that can be explained by regression is also used as a goodness measure. As we can see, our results improve on most prior studies, and the few studies with better results than ours are limited to a small number of courses and much smaller sample sizes, rather than a full cohort of students on different degree courses. We also improve on the goodness of fit given by MANOVA methods for the same data set.

**Table 7:** Comparison of different methods.

Reference	Object of study	Type of method used	Measure of goodness
Current study	1 cohort of 54 degrees in on-campus university	5 ML classification methods	See Tables 4–6
[10]	Several cohorts of 27 degrees in on-campus university	Regression	Explained variance: varying from 73.3% to 83.2%, depending on the area
[11]	1 cohort of 54 degrees in on-campus university	MANOVA	Explained variance: 30%
[17]	2 cohorts of 1 degree in on-campus university	Regression	Accuracy: 88.7%
[20]	2 online courses	Artificial Neural Networks	83.6% and 87.3% for dropout and non-dropout classes, respectively
[22]	5 cohorts of one on-campus university	4 ML classification methods	*F* not provided. Accuracy varying from 86.12% to 87.23%, depending on methods
[23]	1 cohort of 3 degrees in on-campus university	4 ML classification methods	Not numerically but graphically provided. *F*: varying over 40% and below 80% methods
[24]	1 online course	5 ML classification methods	*F* not provided. Accuracy varying from 78.17% to 83.89%, depending on methods
[25]	4 online courses	4 ML classification methods	*F* not provided. Accuracy: 50%-94%. Recall: 20%-90%. Precision: 10.5%-81-8%. Depending on methods
[26]	3 cohorts of online university	3 ML classification methods	*F* varying from 65.65% to 71.91%, depending on methods
[27]	1 online course	4 ML classification methods	F not provided. Recall: 73.9% - 87%, depending on methods

Finally, it is also necessary to underline the good interpretability of the ML-based approach we propose. Based on the output tree, we can build a system of rules that allows us to identify students at risk of dropping out or transferring to another degree. The way the model identifies these students is understandable and interpretable by non-specialist audiences, which has been confirmed. This is a great advantage over other approaches, especially statistical models, which usually require a certain level of expertise to interpret the outputs. Additionally, it is important to note the combination of variables that are considered in the extracted rules, since not only are personal and academic variables highlighted, but the intensity and the modalities of the relationship among these variables are also indicated.

## Conclusions

The results highlight the importance of student services in dropout prevention; as [50] concluded, institutions should pay attention to students’ not only in terms of academic guidance but also in terms of personal counselling and support, involving the education community as a whole [51]. Several studies have contributed to this conclusion, for instance, [52] found that a good relationship between administrative staff and students can help to increase persistence rates. Students’ positive perceptions about institutional support promote engagement and decrease the likelihood of student departure [53]. It is also necessary to point out the need to pay special attention and provide particular guidance to disadvantaged students, regardless of the cause (disabilities, learning difficulties, personal situations, etc.), a commitment that every public institution should make.

Therefore, considering all the factors identified in the analysis, and bearing in mind the research hypotheses, we can conclude that:

- Since rules to predict university dropout were derived from ML techniques, even when considering a whole cohort of students in all degree courses in a medium-sized university, RH1 can be accepted.
- The rules produced do combine personal, academic and environmental variables. Therefore, RH2 is also confirmed.
- Some of the associations revealed by this ML analysis are new in the context of the problem; this leads us to also accept RH3.
- Finally, the improvement of performance produced with this method is also supported by the comparison displayed in Table 7, which confirms RH4.

It was not expressed as a research hypothesis, but results also confirm the improvement of interpretability of results, especially the visual support in Fig 7 which reinforces the idea that ML methods simplify the understandability of the extracted rules, and allow us to identify risky student profiles.

In conclusion, we want to underline the importance of applying this type of technique to an entire university population, (as shown in this study) and not solely to a course, a MOOC, or a single degree. We believe this reinforces the importance of our results, despite producing other problems (such as those indicated for the sample sizes) that should be investigated in the future by using new data from recent years. Also, after analysing the results, we believe that more variables should be added to the model in the future, for instance, the size of the teaching group the students are in, and maybe the size of the faculty (from the data analysed in this paper, this varies from a few hundred students and only one degree in the smallest faculty up to several thousand students and more than seven degree courses in the biggest faculty). The possible effect of the size of this micro-level environment has been noted.

### Limitations and future work

One limitation of this research is that, although the sample size is quite large (especially for this type of study), representing approx. 20% of the cohort, a greater sample size would increase accuracy in predictions, especially in those classes with less representation in the sample (the *Change* class, in particular). In addition, a total probabilistic sampling would enhance the generalization of conclusions, although it is very difficult and extremely rare in wide-range studies with large samples like this one.

An interesting future line of work would be to develop institutional systems that can provide real-time data, making the identification of students at risk of dropout easier, giving appropriate intervention measures at the time to re-engage students in their academic journey [45]. This could be accomplished by implementing detection tools on academic data systems at universities based on real-time data using ML methods, as they have the advantage of learning in real-time and can contribute to the improvement of institutional knowledge management systems [43,54,55]. The integration would improve both the detection method and the effectiveness of the retention policy. Following the traces of the most likely dropout leaves in the trees leads us to identify at-risk student profiles from the persistance point of view, allowing institutions to concentrate additional resources on students with a high risk profile (as suggested in [16]).
